# Refraction without Mydriatics

**Published:** 1896-01

**Authors:** Richard H. Satterlee

**Affiliations:** 189 Delaware Avenue


### REFRACTION WITHOUT MYDRIATICS.

*To the Editor :*

Sir—In further elaboration of my article on the use of mydriatics, permit me to say that no new method was ever advocated that did not bring out expressions of incredulity. Believing in "holding fast to that which is good," many ignore that which is better.

I realise that the weight of accepted authority is against me. Most of the authors have very little time to devote to refraction and the kind of work many of them do is very indifferent. If this was not the case we would not hear so much about muscle cutting as a lucrative substitute for proper refraction.

Mydriatics give the ophthalmologist a false sense of security. Certain cases are on record of ciliary spasm that two weeks of atropia would not relieve. A case at present under my observation has had mydriatics galore by two well-known oculists, both prescribing a minus lens over one eye. I prescribed a plus glass, relieving the head symptoms and obtaining $\frac{20}{20}$ vision.

RICHARD H. SATTERLEE.

189 Delaware Avenue.

---

They are getting after the "vitapaths" in Cincinnati, and very properly. One of them was lately arrested and fined for practising without a license. Judge Dustin said, in pronouncing sentence: "Men who knowingly go into a sick room and prevent anything being done for a dying man by silly incantations and laying on of hands are responsible for his death and ought to be on a par with a murderer in the eyes of the law. God help the dying man who relies upon you or any of the so-called graduates of quackery. You speak of vitapathy being of a higher power than medicine, and you say you ordain ministers at the same time you matriculate vitapathic physicians. Your methods are an insult to intelligence, their practice is a criminal abuse of ignorance and your college a disgrace to civilisation." We congratulate the judge upon his elegant characterisation.—*Atlanta Med. and Surg. Jour.*, Oct., 1895.

